# A new look at an old question: when did the second whole genome duplication occur in vertebrate evolution?

**DOI:** 10.1186/s13059-018-1592-0

**Published:** 2018-11-28

**Authors:** Linda Z. Holland, Daniel Ocampo Daza

**Affiliations:** 1 Marine Biology Research Division, Scripps Institution of Oceanography, University of California San Diego, La Jolla, CA 92093-0202 USA; 2 Department of Organismal Biology, Uppsala University, 75236 Uppsala, Sweden; 3 School of Natural Sciences, University of California Merced, Merced, CA 95343 USA

## Abstract

A recent study used 61 extant animal genomes to reconstruct the chromosomes of the hypothetical amniote ancestor. Comparison of this karyotype to the 17 chordate linkage groups previously inferred in the ancestral chordate indicated that two whole genome duplications probably occurred in the lineage preceding the ancestral vertebrate.

## Introduction

**Fig. 1:**
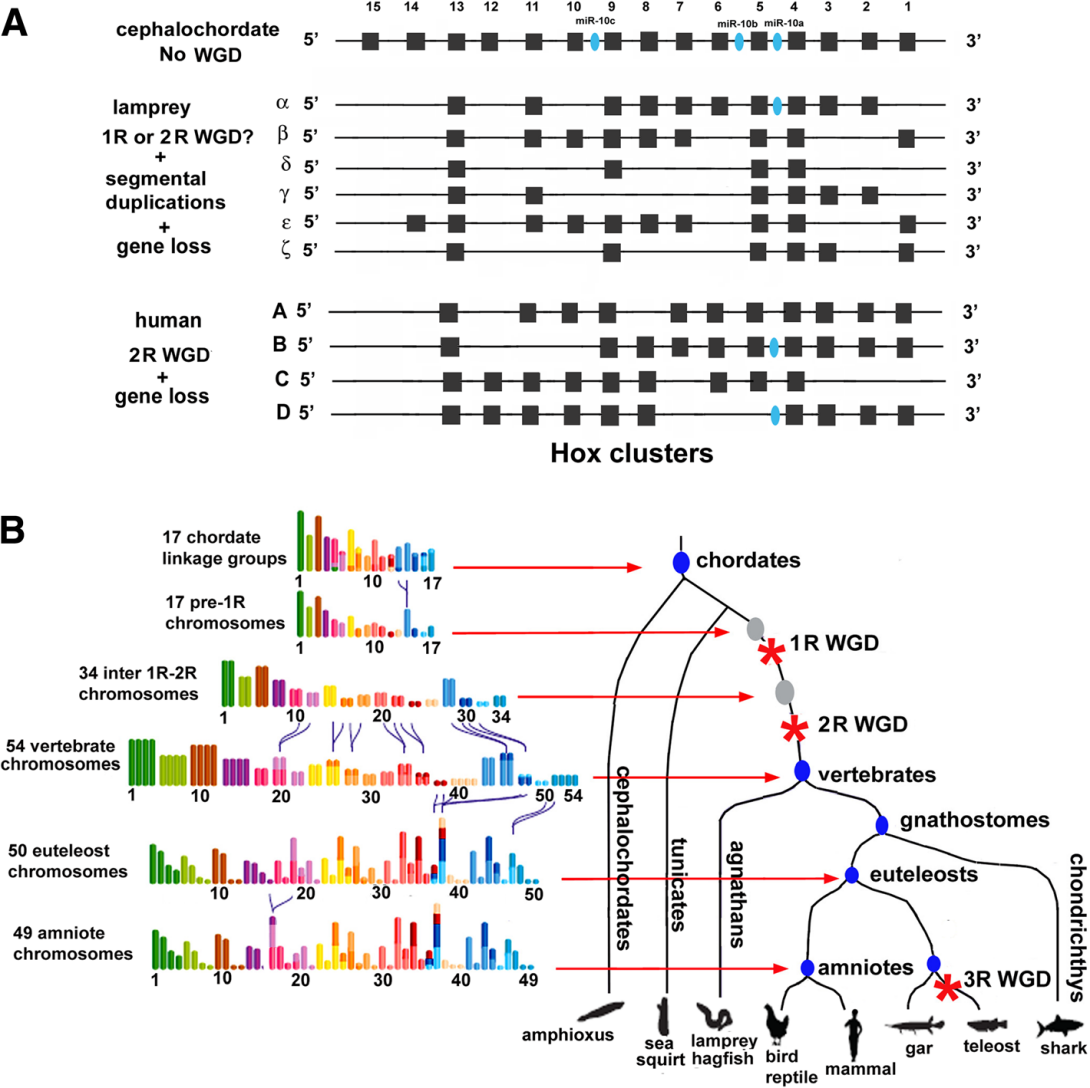
When did the second whole genome duplication (WGD) occur in chordates? **a**. After [4, 8]. Duplications of *Hox* clusters in lampreys and humans are consistent with a single WGD at the base of the vertebrates and a second WGD before gnathostomes evolved. However, the six Hox clusters in agnathans (lampreys and hagfish) are consistent both with WGD at the base of the vertebrates plus duplications of chromosomal segments, together with independent duplications and losses of individual genes, or with two WGDs at the base of the vertebrates plus segmental duplications and individual gene gains and losses. **b**. Adapted from Fig. 5 in [5]. The scheme of chromosome evolution in invertebrate chordates (cephalochordates and tunicates) and vertebrates. In this scheme, the ancestral chordate had seventeen chromosomes as deduced from synteny between the amphioxus and vertebrate genomes [9]. Subsequent to one WGD, a second WGD plus chromosomal fusions (indicated by curved lines) resulted in 54 chromosomes at the base of the vertebrates. Additional chromosome fusions occurred in the lineages leading to euteleostomes (bony vertebrates) and amniotes. A third WGD occurred in the teleost lineage. The heights of the bars representing chromosomes or linkage groups are in proportion to the number of genes located in each one. The colors correspond to those of the 17 pre-1R chromosomes. The size of the colored segments is proportional to the number of genes in each one

Since Susumu Ohno proposed nearly 50 years ago that the genomes of birds and mammals had evolved by tetraploidization, that is, by whole genome duplication (WGD) [1], it has been hotly debated when in evolution these duplications occurred and how many there were. The argument currently revolves around the six clusters of *Hox* genes in agnathan (lamprey and hagfish) genomes compared to four in birds and mammals and one in the invertebrate chordate amphioxus (Fig. 1a). Although it has been proposed that genomes in agnathans and gnathostomes duplicated independently, it is generally thought that at least one genome duplication occurred at the base of the vertebrates. However, opinion has been divided as to whether a second genome duplication also occurred at the base of the vertebrates or separately in gnathostomes and agnathans after their split.

Analyses of lamprey genomes have not definitively resolved this conundrum. One analysis proposed two rounds of WGD at the base of vertebrates followed by a third WGD in lampreys [2]. However, the most recent analyses by Smith et al. favored only one round of WGD at the base of the vertebrates, coupled with chromosome-scale duplications in lampreys [3, 4]. These analyses were based on comparisons of the lamprey, chicken and gar genomes. Six of eleven orthology groups identified in the lamprey corresponded to two derived chicken chromosomes, indicative of only one WGD, while two orthology groups had no clear relationship to chicken chromosomes. Importantly, three of the eleven orthology groups corresponded to four derived chromosomes previously well-cited to support two rounds of WGD; one included *Hox* and *MHC*, a second included *ParaHox* and *NPYR* and the third included *RAR* and *ALDH1*. However, Smith et al. [4] posited a different origin for these. For example, two chromosomes shared more duplications of *Hox* genes than other *Hox-*bearing chromosomes, suggesting that they derived from the duplication of a chromosome segment more recently than the WGD at the base of the vertebrates.

Taken together, the data supported three chromosome-scale duplications, followed by one WGD at the base of the vertebrates, although two WGDs were not ruled out [4]. The data were also compatible with some chromosome fusions and fissions in the gnathostome lineage. In contrast, a recent study by Sacerdot et al. in *Genome Biology* adopted an entirely new approach to address the timing of WGDs in vertebrates and reached a very different conclusion [5].

## Reconstructing the genome of the ancestral amniote

To bridge the large evolutionary distance between extant vertebrates and the ancestral chordate with an unduplicated genome, Sacerdot and colleagues first reconstructed the ancestral amniote genome. To do so, they first applied the AGORA algorithm (Algorithm for Gene order Reconstruction in Ancestors) that they had previously developed [6] to the gene orders, orientations and gene trees of 61 genomes of extant animals from the Ensembl database. These included 40 mammals, 3 birds, 2 reptiles, 1 amphibian, 8 teleosts, 1 coelacanth, 2 tunicates, 1 nematode and 1 fly. Unfortunately, Ensembl does not include the genome of any cartilaginous fish (e.g. the elephant shark, *Callorhinchus milii*, a chimaera, which has the slowest-evolving vertebrate genome known), or those of hemichordates, echinoderms or cephalochordates – invertebrate deuterostomes whose genomes have not undergone the considerable gene loss and compaction characteristic of tunicate genomes.

Sacerdot et al. then identified putative ohnolog gene pairs in this ancestral amniote genome and produced sets of Contiguous Ancestral Regions (CARs). Ohnologs are homologous genes within a species resulting from WGDs. Finally, they grouped these CARs into a set of 51 that significantly fell into 17 groups of four, or tetrads; the expected outcome of two rounds of WGD. To distinguish the pattern of chromosomal fusions and fissions during evolution, they wanted to compare these 17 tetrads to the organization of genes in an unduplicated pre-vertebrate genome. However, tunicate genomes are evolving very rapidly and are quite divergent from those of other invertebrate deuterostomes, while the published genome assembly of the cephalochordate *Branchiostoma floridae* is overly fragmented for the analysis. Therefore, the authors compared their 17 tetrads of CARs to the 17 ancestral chordate linkage groups previously determined by comparison of the synteny of genes on human chromosomes and on scaffolds of the *B. floridae* genome. Remarkably, each of these chordate linkage groups correlated with one predominant CAR tetrad. They concluded from this comparison that the genome of the ancestral vertebrate had 17 chromosomes that then duplicated into 34 chromosomes through one round of WGD (1R). Subsequently, there were 7 chromosome fusions resulting in 27 chromosomes, which at the origin of the vertebrates, before the agnathan/gnathosome split, duplicated again (2R WGD) into 54 chromosomes. After the 2R WGD, there were four additional fusions before the ancestral bony vertebrate followed by a fifth fusion before the base of the amniotes. Thus, the ancestral karyotype of bony vertebrates included 50 chromosomes and the ancestral amniote had 49 chromosomes (Fig. 1b).

In addition to reconstructing duplications, fusions and fissions of chromosomes during evolution, the AGORA algorithm can calculate the gene orders on the chromosomes of the hypothetical amniote ancestor by comparing the gene orders on the chromosomes of its extant descendants [6]. This reconstructed ancestral amniote genome contains 80% of the 15,854 genes in the CARs. The uneven distribution of these genes on the 49 chromosomes of the ancestral amniote could reflect reality or alternatively the difficulty of reconstructing the gene order on chromosomes that had undergone considerable gene rearrangement during evolution.

Even so, two things stand out in the analysis. The first is that comparison of the ancestral amniote genome with the CARs grouped into 17 tetrads to the super-scaffolds of the lamprey (*Petromyzon marinus*) genome assembly shows a clear 1-to-four pattern consistent with 2R WGD *before* the agnathan/gnathostome split. This is at odds with the results of Smith et al. [3, 4] who found strong evidence for only 1R WGD in the lamprey when compared to the chicken. This discrepancy could be explained by the inclusion of genomic data from more species in reconstructing the hypothetical ancestral amniote genome, a process that in itself also eliminates confounding genome rearrangements that have occurred in vertebrates subsequent to the amniote ancestor. It is likely also due to the inclusion of phylogenetic data to determine the CAR tetrads, thus revealing ohnologs that diverged at the base of vertebrate evolution.

The second result that stands out is the correspondence between gene order on human chromosomes and the 17 pre-1R chromosomes. When the 17 pre-1R chromosomes are color coded (Fig. 1b) and the colors of each gene transferred to the positions of the 8282 human genes that descended from the pre-1R genes, the pattern of genome duplications and translocations is apparent. For example, chromosome 1 in the pre-1R genome contains *Hox* genes. A large segment of human chromosome 2 contains the *HoxD* cluster plus many other genes corresponding to those on pre-1R chromosome 1. The other 3 human *Hox* clusters are located on human chromosomes 7, 12 and 17. In addition, asubstantial numbers of homologs of other genes on pre-1R chromosome 1 are located on human chromosomes 1, 3, 10, 16 and 22, indicative of translocations.

## Concluding remarks

The study by Sacerdot and colleagues [5] may be the best estimate yet of the history of chromosome duplication, fusion and fission in early vertebrate evolution, although a definitive answer as to whether the second WGD occurred at the base of the vertebrates or after the agnathan/gnathastome split may prove elusive. The reconstruction of the genome of the hypothetical amniote ancestor was based on the genomes of 2 ascidian tunicates, 1 fruit fly,1 nematode, 1 agnathan and 56 gnathostomes. The tunicate and nematode genomes are evolving rapidly and secondarily reduced with considerable gene loss (e.g. only 9 Hox genes in *Ciona*) and the fruit fly has a high proportion of rapidly evolving genes. Whether including the genomes of a shark and more slowly evolving invertebrate deuterostomes such as cephalochordates and echinoderms or more agnathans would have changed the outcome of the analysis is, of course, unknown. The inclusion of more agnathan genomes (e.g. those from the Southern hemisphere) could also help distinguish chromosome-scale events that occurred before the agnathan/gnathostome split from those that occurred independently in agnathans. The fact that both lamprey and hagfish genomes appear to have six Hox clusters [7], indicates that they will not provide the final solution. It is unfortunate that these are the only extant agnathans and that time machines exist only in fiction. Therefore, the argument as to when the second WGD occurred may never be entirely settled.
